# Conserved G-Quadruplex-Forming Sequences in Mammalian *TERT* Promoters and Their Effect on Mutation Frequency

**DOI:** 10.3390/life13071478

**Published:** 2023-06-29

**Authors:** Vera V. Panova, Nina G. Dolinnaya, Kirill A. Novoselov, Viktoriia Yu. Savitskaya, Ivan S. Chernykh, Elena A. Kubareva, Andrei V. Alexeevski, Maria I. Zvereva

**Affiliations:** 1 Department of Bioengineering and Bioinformatics, Lomonosov Moscow State University, Leninskie Gory 1, Moscow 119234, Russia; verapanova877@gmail.com (V.V.P.); iv.chernykh@yandex.ru (I.S.C.); 2 Department of Chemistry, Lomonosov Moscow State University, Leninskie Gory 1, Moscow 119991, Russia; dolinnaya@hotmail.com (N.G.D.); kir98alekc@mail.ru (K.A.N.); svk1896@mail.ru (V.Y.S.); maria.i.zvereva@yandex.ru (M.I.Z.); 3 Belozersky Institute of Physico-Chemical Biology, Lomonosov Moscow State University, Leninskie Gory 1, Moscow 119992, Russia; aba@belozersky.msu.ru; 4 Department of Mathematics, Scientific Research Institute for System Studies, Russian Academy of Sciences, Nakhimovskii Prospekt 36-1, Moscow 117218, Russia

**Keywords:** *hTERT* promoter, G-quadruplex, bioinformatics analysis, orders of mammals, nucleotide substitution, block alignment, mutation frequency

## Abstract

**Simple Summary:**

Guanine-rich genomic DNA sequences are known to be able to fold into noncanonical G-quadruplex (G4) polymorphic structures. G4s play important physiological roles in DNA replication, repair, mutagenesis, regulation of gene expression, etc. G4-forming DNA sequences (G4 motifs) predominate in the regulatory elements (promoters) of human oncogenes, which indicate G4 involvement in cancer progression. The object of our study was the search for G4 motifs located in the promoter regions of the telomerase reverse transcriptase (*TERT*) oncogene, the product of which is responsible for the immortalization of cancer cells. Using a combination of known and newly developed bioinformatics approaches, we identified G4 motifs in the *TERT* promoter regions of 141 mammalian species belonging to 20 orders, 5 of which, including primates and predators, contain more than 10 species. Comparison of their location in the *TERT* promoters, degree of conservation, and mutability potential revealed that the G4 motifs of the *TERT* promoters across the mammalian class are evolutionarily conserved and are therefore biologically significant. The obtained data support our hypothesis that G4s can interfere with DNA repair pathways and affect the evolutionary adaptation of organisms and species.

**Abstract:**

Somatic mutations in the promoter region of the human telomerase reverse transcriptase (*hTERT*) gene have been identified in many types of cancer. The *hTERT* promoter is known to be enriched with sequences that enable the formation of G-quadruplex (G4) structures, whose presence is associated with elevated mutagenicity and genome instability. Here, we used a bioinformatics tool (QGRS mapper) to search for G4-forming sequences (G4 motifs) in the 1000 bp *TERT* promoter regions of 141 mammalian species belonging to 20 orders, 5 of which, including primates and predators, contain more than 10 species. Groups of conserved G4 motifs and single-nucleotide variants within these groups were discovered using a block alignment approach (based on the Nucleotide PanGenome explorer). It has been shown that: (i) G4 motifs are predominantly located in the region proximal to the transcription start site (up to 400 bp) and are over-represented on the non-coding strand of the *TERT* promoters, (ii) 11 to 22% of the G4 motifs found are evolutionarily conserved across the related organisms, and (iii) a statistically significant higher frequency of nucleotide substitutions in the conserved G4 motifs compared to the surrounding regions was confirmed only for the order *Primates*. These data support the assumption that G4s can interfere with the DNA repair process and affect the evolutionary adaptation of organisms and species.

## 1. Introduction

According to recent studies, the DNA G-quadruplexes (G4s), one of the most widely investigated non B-form DNA structures, are considered to be an integral part of complex regulatory systems in both normal and pathological cells. G4s are four-stranded nucleic acid secondary structures formed from guanine-rich sequences by the stacking of two or more G-tetrads, planar arrangements of four guanine residues stabilized by Hoogsteen hydrogen bonding and coordination to a central cation. They can adopt many different topologies depending on how the primary DNA structure folds into quadruplex arrangements. In recent years, new tools have been developed to introduce some improvements in the prediction of the G4-forming DNA sequences (G4 motifs) throughout the genomes [1]. They radically differ from the standard and most commonly used Quadparser algorithm G_n_L_1-7_G_n_L_1-7_G_n_L_1-7_G_n_, where G_n_ corresponds to G-tracts with n consecutive guanines (n ≥ 3), and L is a semi-arbitrary 1-7-nucleotide sequence corresponding to quadruplex loops; this tool is based on a simple folding rule representing four runs of guanosines separated by relatively short loops. Bioinformatics analysis has shown that G4 motifs are widely represented in the genomes of all organisms, although they are predominantly enriched in eukaryotic genomes. Thus, 95 families of G4 motifs have been identified in the human genome [2]. They often cluster at key regulatory elements and provide a significant potential repertoire for the formation of short-lived G4 structures that influence essential biological processes such as DNA replication, telomere maintenance, gene expression, recombination, and DNA repair, as well as genetic and epigenetic integrity [3]. Thus, G4s may contribute to replication-dependent genome instability due to replication fork arrest [4,5,6] and increase the frequency of mutations, deletions, and recombination events [7,8]. G4s represent a strong block for replication and transcription, not only by themselves, but also due to oxidative lesions, which are especially susceptible to DNA regions containing a large number of guanine bases [9,10]. It is well known that DNA oxidation occurs mainly at guanines, since they have the lowest redox potential among all native nucleobases [11].

Given that G4 structures alter DNA accessibility to damaging agents and affect the efficiency and accuracy of DNA repair pathways [12], they are subjected to natural selection and may be the driving force behind genome evolution [13]. It can be assumed that it is these noncanonical structures that switch the activity of genes that ensure the development of *Vertebratas* during the transition from gill to pulmonary respiration. At the same time, evolutionary conservation of G4 motifs located in certain gene regions should be observed if related G4 structures are biologically relevant.

Here, we used bioinformatics tools to analyze the presence and variability of G4-forming sequences in the promoter regions of the *TERT* gene for 158 mammalian species. The human TERT protein (hTERT) is the catalytic subunit of telomerase, an enzyme complex that is primarily responsible for maintaining telomere length and replication potency of stem cells [14]; its activity is thought to be vital for the immortalization of cancer cells [15]. The core region of the *hTERT* promoter (approximately −180 ÷ +1 from the transcription start site (TSS)) contains a 68 nt G-rich site with twelve consecutive G-tracts, which have been shown to fold into three stacked parallel-stranded G4s [16]. Given the key role of the TERT enzyme and the obvious importance of telomere biology in the life of multicellular animals, it can be assumed that the *TERT* genes are the subject of evolution, reflecting the adaptation of organisms to various living conditions.

The TERT protein contains three canonical domains: (1) a telomerase essential N-terminal domain, (2) a telomerase RNA-binding domain, and (3) a reverse transcriptase domain [17]. Such a structural organization makes it possible to search for putative genes encoding TERT, to determine their promoter regions, and to perform bioinformatics analysis using publicly available genomic data sets and specially adapted software for the joint alignment of stable blocks in the promoter regions of *TERT* genes across different orders of mammals.

The main goal of our study was to test whether the G4 motifs of the *TERT* promoters in the mammalian class are evolutionarily conserved and therefore biologically significant. We searched for G4 motifs separately in the coding and noncoding strands of the *TERT* promoter regions for mammalian species belonging to different orders. This was carried out to determine whether potential G4 structures are involved in the regulation of gene expression at the transcriptional level (if G4 motifs are located in the non-coding strand) or whether they are an element of possible translational regulation if G4 motifs are located in the coding strand and, subsequently, in the RNA transcript. We also analyzed the type and frequency of nucleotide substitutions causing the genome mutations in conserved regions of *TERT* G4 motifs.

## 2. Results

### 2.1. Selection of Mammalian TERT Promoters

Initially, we downloaded data on *TERT* genes from 158 mammalian species available in NCBI Orthologs at https://www.ncbi.nlm.nih.gov/ (accessed on 13 August 2022) For all the *TERT* genes studied, we found a putative transcription start site (TSS) and downloaded the 1000 bp upstream region. We call it the “promoter region” because it contains the core *TERT* promoter, a proximal promoter part containing recognition sites for many transcription factors and a more distant hypermethylation oncological region implicated in cancer progression. After a revision aimed at removing promoter regions with unreliable sequencing results and erroneously determined coordinates, 141 *TERT* genes and their promoter regions remained.

### 2.2. Search Tools for G4 Motifs

To detect G4 motifs in *TERT* promoter regions, we tested three tools with different algorithms: G4Hunter, pqsfinder, and QGRS mapper. Our choice of tool was based on the ability to compare matches to identify conserved G4 motifs. Pqsfinder [18] was developed to search for G4 motifs that allow for imperfect quadruplex formation with potential single bulges and mismatches in the quadruplex core and with a wider range of G-tract lengths and loop sizes. G4Hunter [19] takes into account G-richness and G-skewness (G/C) asymmetry between complementary DNA strands of a given sequence and uses simple encoding and sliding window statistics that can account for various types of quadruplex defects. As a result, it provides a score (propensity to form G4 structure). It is not clear how to compare such regions in different promoters. QGRS Mapper [20] provides a pattern search: G_3+_ L_1-30_ G_3+_ L_1-30_ G_3+_ L_1-30_ G_3+_.

Since the search results of three different G4 predictors varied significantly, we chose the QGRS mapper as the main tool. Using the QGRS mapper, we can lose a number of G4 motifs that possibly form defective G4 structures. On the other hand, we have the ability to easily compare matches in G-tracts and to detect substitutions within loops of conserved G4 motifs. In addition, the pqsfinder and G4Hunter programs were rejected because they identify G4 motifs with dinucleotide G-tracts that do not provide the formation of a thermodynamically stable G4 structures. Additional constraints adopted in our study: (i) the maximal total length of the G4 motif does not exceed 45 nucleotide units; (ii) each G-tract must fit into the sequence window defined by the starting position of the first G-tract; (iii) each subsequent G-tract must lie behind the 3′-end of the previous one (without overlap). All of these options can be freely configured using the QGRS mapper package.

### 2.3. Distribution of G4 Motifs in TERT Promoters across the Mammalian Class

The search for G4 motifs was carried out in the *TERT* promoter regions of 141 mammalian species belonging to 20 orders, 5 of which are *Primates* (primates), *Artiodactyla* (artiodactyls), *Carnivora* (predators), *Chiroptera* (bats), and *Rodentia* (rodents) and which contain more than 10 species. All other orders were considered together. A list of mammalian species used in this study is shown in Table S1. Figure S2 demonstrates taxonomictrees for five orders of mammals.

At the first stage, we compared histograms of distances (the number of nucleotide units) between a certain G4 motif and TSS on the coding and noncoding strands of the 1000 bp *TERT* promoter regions for all studied species of mammals. The NCBI gene database used in the work indicates which of the *TERT* gene strands contains the coding sequence and which is complementary to it (i.e., is noncoding or template).

**Figure 1:**
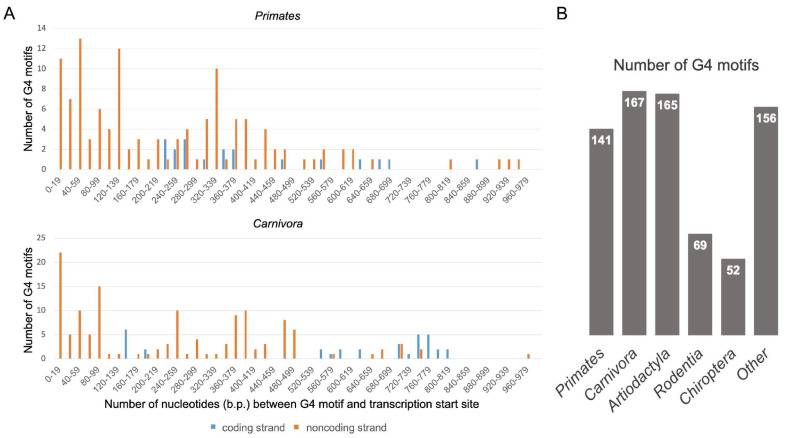
Distances of G4 motifs from the TSS on the coding (blue columns) and noncoding (orange columns) strands of the 1000 bp *TERT* promoter region for *Primates* and *Carnivora* (**A**). The number of found G4 motifs for all studied orders of mammals (**B**).

Figure 1A shows examples of histograms obtained for *Primates* and *Carnivora* (similar histograms for other orders of mammals are shown in Figure S1); the number of found G4 motifs for all studied orders is listed in Figure 1B. Numerical data on the localization of G4 motifs in the *TERT* promoter zones 0–180, 181–500 and 501–1000 bp from TSS are presented in Table 1.

Only 6 out of 141 mammalian species tested do not contain G4 motifs. The absence of G4 motifs is observed in four rodent and one bat species that are cancer resistant. Diversity in the G4 motif distribution in different orders of mammals is shown in Table 1. In general, the total number of G4 motifs on the noncoding strand of *TERT* promoters is significantly higher than on the coding strand, this effect being more pronounced for the proximal parts of the promoter than for the distal one. In addition, just the core and proximal parts of the *TERT* promoters (~0–400 bp) are enriched in G4 motifs compared to the distal part.

We also estimated the average number of all G4 motifs per species for orders of the following parameters were determined in the block: mammals (Figure S3) and revealed that it strongly depends on the order type.

### 2.4. The Search for Conserved G4 Motifs in the TERT Promoter Regions of Five Mammalian Orders

Although the TERT protein is widely ubiquitous across mammals, not much is known about whether the G4 motifs of *TERT* promoters are conserved in the mammalian kingdom through their evolutionary history.

Note that a multiple alignment of any set of G4 motifs with the accepted restrictions has at least twelve identical columns corresponding to four G-tracts with three consecutive guanosine residues. To avoid false positive predictions of conserved G4 motifs, we used a step-by-step procedure. First, we found blocks of reliable alignments in the promoters of each of the five orders of mammals using the Nucleotide PanGenome (NPG) explorer program (https://github.com/npge/npge, accessed on 13 August 2022). In this work, NPG-explorer input promoters instead of genomes and G4 motif coordinates instead of gene coordinates. The output was a set of alignments consisting of fragments of several (not necessarily all) promoters with more than 80% identical columns and at least 100 columns in length. Using this tool, we performed a cross-species comparison of all found G4 motifs, containing no more than 45 bp, in both coding and noncoding strands of *TERT* promoter regions. The numbers of aligned blocks and *TERT* promoter fragments in each block are shown in Table S2. Clusters of the G4s motifs with intersecting coordinates within the block alignment were found in the blocks. The procedure was carried out separately for the species of each mammalian order. Groups of conserved G4 motifs were identified after visual examination of all clusters.

**Figure 2:**
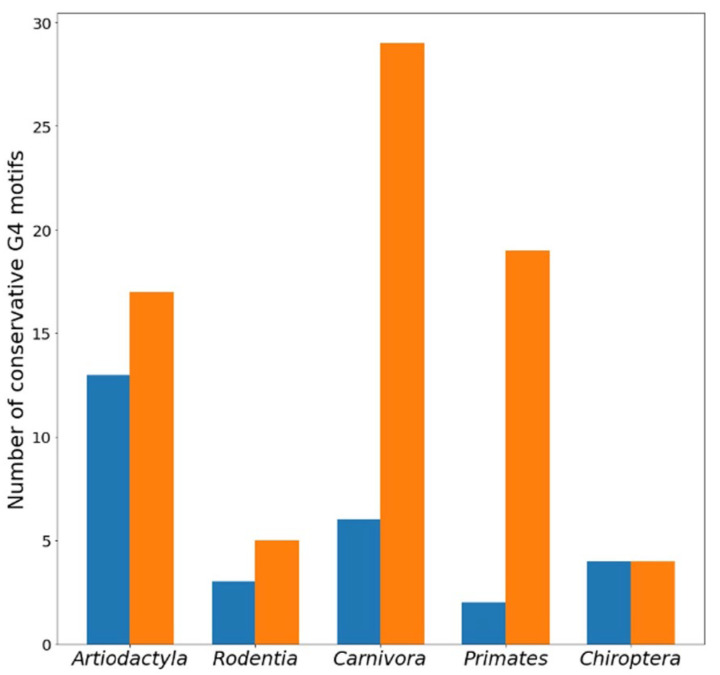
The number of conserved groups of G4 motifs on the coding (blue columns) and noncoding (orange columns) strands of the *TERT* promoter depending on the type of mammalians order.

The numbers of conserved G4 motifs located in different *TERT* promoter zones in coding and noncoding strands for five mammalian orders are presented in Table 2. Comparison of the data in Table 1 and Table 2 shows that the proportion of conserved G4 motifs in relation to all found ones across the compared set of mammalian species depends on the type of order, varying from 18/22% (coding/noncoding strands) for *Carnivora* to 13/11% for *Rodentia*. As can be seen, conserved G4 motif loci lie primarily on the noncoding strand of *TERT* promoter regions (Figure 2), although strand preference is mammalian order specific and correlates with strand-dependent arrangement of G4 motifs (Table 1). Most (79 out of 102) conserved G4 motifs are located in the core and proximal promoter zones. 

**Figure 3:**
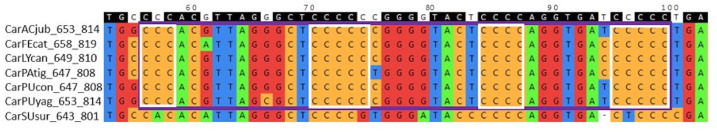
Group of conserved G4 motifs for 6 species (out of 7 examined) of the order Carnivora (predators) in block h7x162. The group is framed in purple. Here, G4 motifs are located in the noncoding strand of *TERT* promoters. In block alignments, promoter fragments are presented on the coding strands, since nucleotide substitutions in conserved regions are usually indicated on the coding strand. That is why the G-tracts of the G4 motifs looks as “C-tracts”; they are shown in the white frames. The consensus sequence is shown in the top line. The species codes and coordinates of fragments taken into alignment in promoters are given in the left. For more detailed explanations, see Materials and Methods, Section 4.2 and Section 4.3.

An example of a group of conserved G4 motifs in the block alignment of *TERT* promoters for seven species of the order *Carnivora* is shown in Figure 3.

We then compared the coordinates of the conserved G4 motifs of the *TERT* promoter regions with the binding sites of several known transcription factors: Sp1, Ets, c-Myc, etc. It was shown that G4 motifs from 23 primate species overlap with the first and second binding sites of Sp1 and with Etsrecognition site immediately before the TSS (Figure S4). The recognition sites of both transcription factors are conserved in the TERT promoters of 23 primate species studied. The Ets site islocated in the loop of conserved G4 motifs. We intend to continue studying this phenomenon in other orders of mammals.

**Figure 4:**
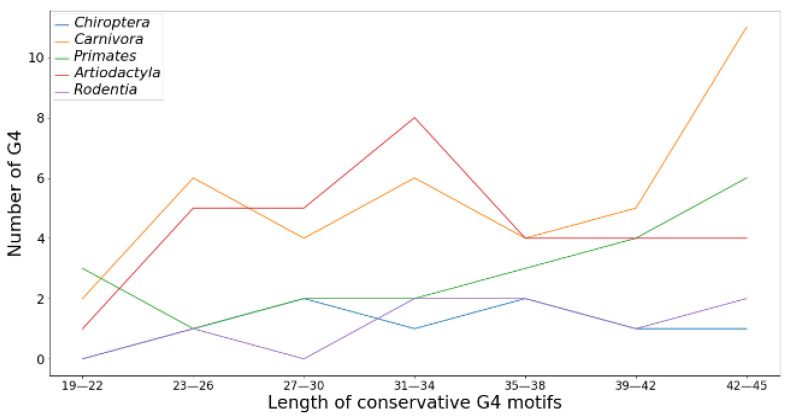
Length distribution of conserved G4 motifs of *TERT* promoters among five orders of mammals.

The length of the conserved G4 depends on the type of mammalian order (Figure 4). As can be seen from the obtained data, species of the order *Carnivora* have an increased number of conserved G4 motifs in *TERT* promoters 40 or more nucleotides in length, while 31–34 nt conserved G4 motifs are most represented in *Artiodactyla*. In mammals of the order *Rodentia* and *Chiroptera*, the distribution of conserved G4 motifs along the length is more or less uniform, without pronounced maxima and minima.

### 2.5. Single-Nucleotide Variants in Groups of Conserved G4 Motifs in the TERT Promoter Regions of Five Mammalian Orders

Since G4 DNA contributes to regulating multiple essential cellular processes, its sequence motifs should evolve under natural selection. For estimating the diversity in G4 motifs of the *TERT* promoters from different orders of mammals, we also used a block alignment approach based on the NPG-explorer program. Nucleotide substitutions can be reliably identified only within groups of conservative G4 motifs. Comparisons of a unique predicted G4 motif within a block alignment with homologous fragments of a different species within a block that do not contain the predicted G4 motifs or with predicted G4 motifs that only partially overlap with the G4 motif in question are less reliable.

**Figure 5:**
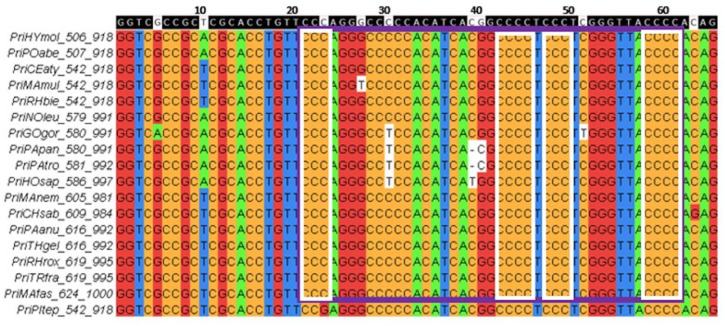
Distribution of nucleotide difference points in the group of conserved G4 motifs for 17 species (out of 18 examined) of the order *Primates* within the block h18x413 were indicated in the coding strand of *TERT* promoter regions (see legend to Figure 3). The group of conserved G4 motifs is in the purple frame. Nucleotides of the coding strands that make up “G4 motifs” (four C-tracts) are framed in white. The consensus sequence is shown in the top line. Three loops of G4 motifs are located between the white frames. Nucleotides marked in white are not identical to consensus, i.e., they are caused by a mutation. There were at least six point mutations in this conserved group of G4 motifs. The species codes and coordinates of fragments taken into alignment in promoters are given in the left.

Point mutations throughout the coding strand of G4 motifs located in *TERT* promoters for 18 species of the order *Primates* are shown in Figure 5 as an example.

**Figure 6:**
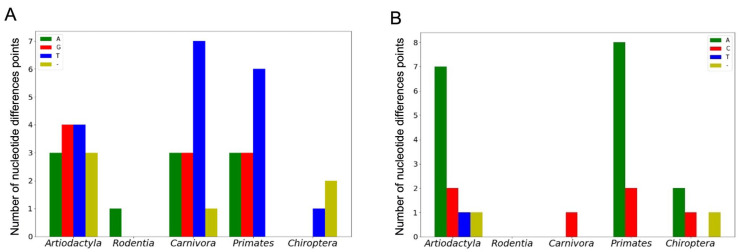
The number of single-nucleotide variants in C-tracts, when another nucleotide is found instead of C (**A**), and in G-tracts, where the G residue is replaced by any nucleotide (**B**). Data were obtained for five orders of mammals.

We separately considered nucleotide substitutions in G4 structure-disruptive positions (occurring in G-tracts) and positions corresponding to G4 loop-forming sequences. Since genome nucleotide substitutions leading to point mutations are usually indicated on the coding strand, the structurally disruptive positions correspond to C-tracts if the G4 motif lies on the noncoding strand and to G-tracts if the G4-forming sequence is located in the coding one. Figure 6A,B shows the number of nucleotide difference points in C- and G-tracts, respectively, depending on the type of mammalian order.

As can be seen, the number and variability of nucleotide difference positions in both C- and G-tracts are taxon-dependent, and C↔T/G↔A substitutions are the most frequent, although a wider set of single-nucleotide variants occurred when G4 motifs are located on the noncoding strand (Figure 6A).

**Figure 7:**
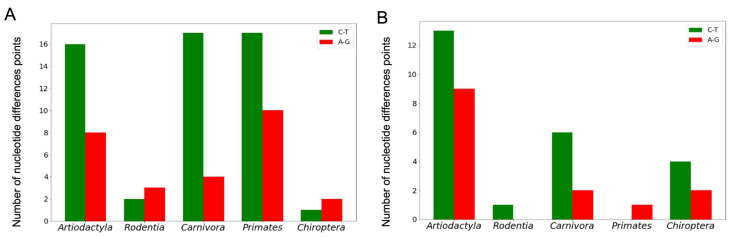
Number of A↔G and C↔T substitutions in the loop-forming sequences of the conserved *TERT* promoter G4 motifs on noncoding (**A**) and coding (**B**) strands for five orders of mammals.

The number of nucleotide difference points and the type of nucleotide substitutions throughout quadruplex loop-forming sequences in conserved G4 motifs were also evaluated in five orders of mammals. Table S3 summarizes these data for the coding and noncoding strands of the *TERT* promoter regions. Based on these data, it can be concluded that the A↔G and C↔T substitutions are the most frequent. Their distribution in the loop-forming sequences of the conserved *TERT* promoter G4 motifs among different mammalian orders is shown in Figure 7.

Next, for each group of conserved G4 motifs, we determined the density of nucleotide substitutions as the ratio of substitution number in G4 loop-forming sequences to the total loop length and compared it with the density of nucleotide substitutions of the control sequence. As a control, we used the *TERT* promoter region equal in length to the total length of the loops either before the initial G4 motif or after the final G4 motif, which did not intersect with any G4 motifs either in the same or in any other blocks. Comparison of the calculated densities of nucleotide substitution for five orders of mammals revealed a higher density of single-nucleotide variants in G4 motifs compared to regions lacking these motifs in each of the studied mammalian order (Table 3).

To evaluate the statistical significance of this statement, we used paired Wilcoxon signed rank test. As a rule, the established difference between the compared parameters is statistically significant when the *p* value ≤ 0.05 [21]. According to the data presented in Table 3, after the application of the Holm–Bonferroni correction, only the order of *Primates* meets this criterion.

## 3. Discussion

The trigger for a detailed bioinformatics analysis of whether the G4 motifs of the *TERT* promoters are evolutionarily conserved across the orders of mammals was the data on G4-driven genomic instability in the *hTERT* promoter region associated with the development of oncological diseases. It has been recently shown by chemical probing and spectroscopic methods that 96 nt DNAs modeling the G-rich strand of the *hTERT* promoter and its variants with G > A point substitutions corresponding to somatic driver mutations fold into three stacked parallel G4s with a local destabilization at the substitution site [16]. Mutations in the *hTERT* promoter play a critical role in the immortality of cancer cells [15] and increase clinical diagnostic potential in thyroid cancer [22], melanoma [23], glioblastoma [24], and bladder cancer [25]. One of the reasons for the selective advantage of malignant cells is the recruitment of a transcription factor (multimeric GA-binding protein, GABP), whose interaction with the mutated promoter provides a mechanism to overcome the downregulation of *TERT* expression and replicative senescence [26,27].

Previously, functional genetic studies have identified several cancer-specific point mutations within the *hTERT* core promoter region [28]. However, not much was known about the role of G4 structures in mutagenesis and how conserved G4 motifs are between mammalian species and orders.

In our work, we used bioinformatics analysis to determine the distribution of G4 motifs in *TERT* promoters, as well as the stability of their composition and sequence across mammalian species of different orders, since the risk of developing cancer is better described in mammals [29]. Some species were removed because their sequences of interest contained more than 100 unread nucleotides or they were not aligned with their closest relatives by the blast2seq service. The search for G4 motifs was performed in the *TERT* promoter regions of 141 mammalian species using the QGRS mapper software program; the size of the analyzed regions was taken as equal to 1000 bp from TSS. The diversity in the distribution of the *TERT* G4 motifs in different orders of mammals (Table 1) is an additional argument in favor of the evolution of *TERT*-mediated regulation. Despite the ubiquity of G4 motifs in promoters of eukaryotic oncogenes [30], their presence is not universal. G4 motifs were not found in six mammalian species (Table 1), including four rodent species and one species of bats that are cancer resistant [31]. This conclusion is consistent with the proposed involvement of G4 structures in the malignant transformation of eukaryotic cells. In other cases, G4 motifs were found to be predominantly located in the region proximal to the TSS (up to 400 bp) and are over-represented on the noncoding strand of the *TERT* promoters, although the choice of strand depends on the order of mammals (Figure 1 and Figure S1, Table 1). The higher density of G4 motifs on the noncoding versus the coding strand suggests that G4s regulate gene expression at the transcriptional rather than the translational level. The latter is possible only if the G4 motifs are located in the RNA transcript [32].

To search for conserved G4 motifs in *TERT* promoter regions of different mammalian species, we applied for the first time a block alignment approach using the NPG-explorer (the number of aligned blocks and *TERT* promoter fragments in each block is indicated in Table S2) and showed that *TERT* promoter sequences capable of folding into G4 structures are evolutionarily conserved throughout the class *Mammalia* (Figure 2 and Figure 3, Table 2) and are therefore biologically relevant. The proportion of conserved G4 motifs in relation to all found G4 motifs across the compared set of mammalian species depended on the type of mammalian order and varied from 11 to 22% (Table 1 and Table 2). The conserved G4 motif loci lie mainly on the noncoding strand of *TERT* promoter regions in the core and proximal promoter zones (Figure 2 and Figure 4). Thus, the formation of G4 may prevent binding of the RNA polymerase complex or transcription factors to the promoter.

In recent years, the deleterious effects of G4s on genome integrity and their potential role in regulating the DNA repair machinery have become the subject of intense research [12]. Recent findings suggest that G4 structures, in collaboration with various specialized proteins, lead to genetic instability, alter the accessibility of DNA to damaging agents, and affect the efficiency and accuracy of DNA repair pathways. In a series of our studies, we have experimentally shown that G4 formation can have a negative impact on DNA mismatch repair that leads to a high mutation rate in G/C-rich regions of oncogene promoters [16,33]. In this study, we found the most frequent nucleotide substitutions (C↔T and G↔A) at positions disrupting G4 structure (occurring in G-tracts) and positions corresponding to G4 loop-forming sequences (Figure 6 and Figure 7, Table S3) and showed that a statistically significant higher frequency of nucleotide substitutions in the conserved G4 motifs of the *TERT* promoters compared to the surrounding regions was only confirmed for the order Primates (Table 3). These data support the hypothesis that the high mutation frequency in the G/C-rich promoters of human oncogenes may be associated with quadruplex-controlled changes in the function of repair proteins.

## 4. Materials and Methods

### 4.1. Selection of Promoter Regions of Mammalian TERT Genes

#### 4.1.1. Initial Data Set

We downloaded the 1000 bp *TERT* promoter regions preceding the TSS (coding strands) for 158 mammals from the COG database dated 13 August 2022 (https://github.com/nooroka/conserved_quadruplexes, accessed on 13 August 2022). In many cases, the TSS coordinates are not explicitly displayed in the GenBank entries, and we were forced to define as the most 5’ end of the available mRNA for a particular gene. This method did not guarantee an accurate determination of TSS. In some cases, the selected promoter regions may be shifted by dozens of bp relative to the true TSS coordinates. We compensate for this obstacle using local alignments (see below).

#### 4.1.2. Data Set Verification and Filtration

We removed 12 promoters from the data set due to inappropriate promoter sequences. *TERT* genes whose promoter regions contained more than 100 N-symbols in a row were discarded. We also rejected five *TERT* genes because the promoter region sequences did not have significant matches compared to the promoter region of the closest related *TERT* gene. For this, the NCBI BLAST2Sequences software was used. As a result, we have retained the promoter regions of 141 mammalian species. Their distribution by order is presented in Table 4.

In the Tables and Figures throughout the paper, we abbreviated the name of organisms as follows: three lowercase letters in the order name (e.g., *Primates* in pri), two capital letters in the genus (e.g., *Canis* in CA), and three lowercase letters in the species name. If the species name consists of two words, the three letters of the second word are also added. Examples: *Homo sapiens* shortens to PriHOsap, *Felis catus* to CarFEcat, *Canis lupus dingo* to CarCAlupdin.

### 4.2. Methods for Detecting G4 Motifs in TERT Promoter Regions

After a preliminary comparison of three existing programs, G4Hunter, pqsfinder and QGRS mapper, we chose QGRS mapper.

#### 4.2.1. G4Hunter

To find G4-like motifs, the G4Hunter algorithm calculates a score for each sliding window based on the ratio of G and C (G-richness) and the distribution of G/C between complementary strands (G-skewness). G-tracts, their length and the length of the loops between them are not controlled and are not presented in the output data [19]. We used G4Hunter to search for G4 motifs in *TERT* promoters with sliding window parameters of 25, score 1, and 1.25. Among the G4 motifs found were G-tracts composed of two guanosines.

#### 4.2.2. Pqsfinder

The pqsfinder algorithm was created to search for various G4 motifs, including those with insertions and mismatches within G-tracks. We used pqsfinder to search for G4 motifs in *TERT* promoters with parameters of minimum score = 30, maximum length = 45, and allowable defects = 3. Among the G4 motifs found were G-tracts composed of two guanosines. The G4 motifs detected by both pqsfinder and G4Hunter may provide a more complicated G4 structures than “classical” ones. This is their advantage. To discover conserved G4 motifs, we prefer to deal with predictions of G4 motifs with typical sequences of G-tracts and loops between them. In addition, we do not consider G4 motifs with G-tracts containing two guanosines, which are detected by both pqsfinder and G4Hunter algorithms, since the G4 structures formed by such G4 motifs are not sufficiently stable.

#### 4.2.3. QGRS Mapper

The QGRS mapper, which searches G4 motifs corresponding to the pattern G_3+_ L_1-30_ G_3+_ L_1-30_ G_3+_ L_1-30_ G_3+_, helps to find them in *TERT* promoter regions of different mammalian orders. QGRS mapper v.1 was launched via the web version. Only non-overlapping G4motifs were considered. The maximum sequence length is 45 nucleotides, the minimum length of the G-tract is three consecutive guanosines, and the length of the loop is from 1 to 30 nucleotides. The coordinates of G4 motifs were determined on the coding DNA strand. To reach our goal of finding conserved G4 motifs and nucleotide substitutions within them, we chose QGRS mapper.

### 4.3. Detection of Conserved G4 Motifs

We searched for conserved G4 motifs in selected *TERT* promoter regions separately for each of five mammalian orders containing more than 10 species. A preliminary test showed that all found reliable local alignments consist only of promoter fragments of the same order. Conserved G4 motifs were detected in two steps.

First, we created reliable local alignments among the promoter regions of each mammalian order. For this, we used the NPG-explorer program (https://github.com/npge, accessed on 13 August 2022). The parameters used in reliable blocks (shortened as “block alignments”) are: (i) alignment identity is 80% or more, (ii) block alignment length is 100 positions or more, (iii) the block cannot be extended to any height (adding an extra fragment) nor by width (adding new alignment columns at one or both ends of the alignment). NPG-explorer also detects blocks that do not fit the constraints. In some cases, they were taken into account at the next step.

Second, to detect G4 motifs in different mammalian species with intersecting coordinates within the block alignment, we created the program AlAn, v.1.2.2 (https://github.com/IvanSChernykh/AlAn, accessed on 13 August 2022). This program takes NPG-explorer input–output files and outputs clusters of G4 motifs from promoter regions of two or more species with mutually intersecting coordinates within block alignment. The size of the intersection is an input parameter.

### 4.4. Definition of the Group of Conserved G4 Motifs

**Figure 8:**
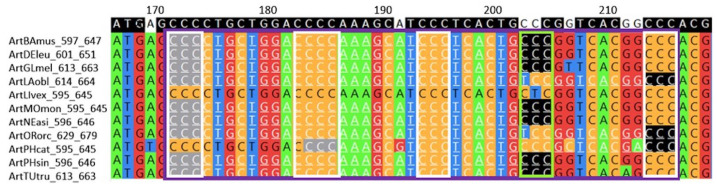
Group of conserved G4 motifs for 11 species of the order *Artiodactyla* within the h11x275 block. The h11x275 block name (in NPGexplorer) consists of the “h” prefix, the number of fragments in the block (11), the “x” delimiter, and the alignment length (275). The sequences of all TERT promoter fragments in any alignment throughout the text are on the DNA-coding strand. Thus, transcription start sites are to the right of the fragments shown. The numbers in the top line are the alignment position numbers. The coordinates of the block fragments in the promoter are shown on the left after the species codes. The coordinate of the G nucleotide in the ArtBAmus promoter fragment at position 170 is approximately 597 + 170 = 767.The exact number depends on the number of gaps in ArtBAmus preceding position 170. The consensus sequence is shown at the top. The found conservative G4 motifs are located on the noncoding DNA strand. Therefore, G-tracts of G4 motifs resemble “C-tracts” and are framed in white. The group of conservative G4 motifs is framed in purple. The QGRS mapper’s algorithm detected G4 motifs separately for each species. The first G-tracts (visible in the alignment as the right-most “C-tracts”) are highlighted in black, and the last G-tracts are highlighted in grey. The first and last G-tracts are not aligned; moreover, the G4 motif is not found at all in the fifth (from top to bottom) sequence. In the three columns framed in light green, G-tracts are found in some sequences and not found in others due to nucleotide substitutions shortening the G-tracts to two guanines. According to our rule, among variants for identifying conserved G4 motifs, the variant with equal coordinates of G-tracts is selected, which includes the maximum number of conserved G4 motifs in the group. This is a purple-framed group with white-framed G-tracts, conserved in all 11 species of block alignment.

We consider G4 motifs within a block alignment to be conservative if their four G-tracts have the same coordinates within the blocks. All clusters of intersecting G4 motifs were considered. Figure 8 shows some of the problems encountered during the test. An expert analyzing local alignment with a cluster of intersecting G4 motifs tries to include the maximum possible number of sequences in the group of conserved G4 motifs by locally shifting the coordinates of G-tracts, observing all accepted restrictions. The simplest example is the selection of three guanine residues (underlined) in the G-tracts of four or more guanines: GGGG or GGGG. The QGRS mapper may choose differently in various promoters. A fragment with a substitution in the G-tract, such as GGGA, allows the GGGG variant to be chosen to include more members in the group of conserved G4 motifs.

There were also more complex situations. We do not yet have an algorithm for elections, because not all imaginary problems appeared in our data.

After revision, conserved G-tracts may include G-tracts of different lengths equal to three or more guanine residues. We adopted this form of description because we see no reason to select three conservative columns of guanines: the first three or the last three, out of four (Figure 8).

### 4.5. Frequencies of Nucleotide Substitutions within G4 Motifs

Substitutions can be reliably identified only within groups of conserved G4 motifs. Comparison of a unique predicted G4 motif within a block alignment with homologous fragments of a different mammalian species that do not contain the predicted G4 motifs or with predicted G4 motifs that only partially overlap with the G4 motif in question is less reliable. First, the prediction of a non-conserved G4 motif corresponds with a low probability to a DNA fragment that forms a G4 structure in the cell. Second, it is difficult to classify substitutions within a sub-alignment in the predicted unique boundaries of the G4 motif: there are too many options, either the G4 motif occurs in only one species, or it is just one G4 motif left over from the G4-forming promoter region in a distant ancestor of all species represented in the block alignment.

#### 4.5.1. Nucleotide Substitutions in G4 Loop-Forming Sequences of Conserved G4 Motifs

According to the definition of groups of the conserved G4 motifs (Section 4.4), we know the boundaries of the three loops between the four G-tracts. At the same time, there are no substitutions in the G-tracts of conserved G4 motifs, by their definition. All substitutions within loops were collected in a file. In each alignment column within the loop, except for the conserved ones, all nucleotides are considered to be substituted with respect to the consensus nucleotide.

#### 4.5.2. Control Data Set for Comparison with Substitution Frequencies in G4 Loop-Forming Sequences of Conserved G4 Motifs

For each group of conserved G4 motifs in the same block of reliable alignment, we selected a sub-alignment with the same set of mammalian species as in the group and the same length as the sum of the lengths of G4 loop-forming sequences that do not intersect with any of the predicted G4 motifs. In this sub-alignment, we calculated the substitution in exactly the same way as we did for loop substitutions.

#### 4.5.3. Comparison of Differences between Nucleotide Substitution Frequencies in Loops of Conserved G4 Motifs and in Control Data Sets

For each group of conserved G4 motifs, the density of loop substitutions was calculated as a ratio of the number of detected substitutions to the sum of loop lengths. This density is compared to the substitution density in the control alignment. The control alignment was chosen within the same block alignment, it contained fragments of the same type as the group of conserved G4 motifs, and the length of the control alignment was equal to the sum of the loop lengths in the group. A control alignment was chosen that did not overlap with any of the predicted G4 motifs; gaps of 3 or more positions were not allowed in the control alignment. In rare cases where a control alignment could not be selected in the same block alignment, it was selected in another block alignment containing the same mammalian species. The number of substitutions within the control alignment was counted, and their density was calculated in the same way as in the loops of the group of conserved G4 motifs.

As a result, we obtained a set of pairs (substitution density in the loops of the group of conservative G4 motifs and substitution density in the corresponding control alignment for each of the five studied orders of mammals). The Wilcoxon test was used to assess the statistical significance of the fact that the density of mutations in G4 motifs is higher than in regions not containing G4 motifs.

## 5. Conclusions

The computational predictions indicate that G4 motifs are predominant in human oncogene promoters compared to the rest of the genome, strongly suggesting a role of G4 structures in cancer progression. In this study, we identified G4 motifs in the *TERT* promoter regions of 141 mammalian species and compared their location, degree of conservation and mutability potential using bioinformatics analysis. In order to address this, we used a combination of known and newly developed approaches. For searching for G4 motifs, we applied three tools: pqsfinder, G4Hunter and QGRS mapper; the latter was chosen as the main one. To identify conserved G4 motifs in the *TERT* promoter regions of five mammalian orders: *Primates* (primates), *Artiodactyla* (artiodactyls), *Carnivora* (predators), *Chiroptera* (bats), and *Rodentia* (rodents), we used for the first time a block alignment approach based on the NPG-explorer. Thus far, nucleotide pangenome software has predicted evolutionary relationship at the nucleotide level between two or more entire genomes. Here, the study focused on comparing functionally important genome regions across a large number of mammalian species. Under the selected similarity criteria (identity of block 80% or more, their length is not less than 100 columns), blocks of significant alignments containing G4 motifs were found in the *TERT* promoters of each of the five mammalian orders. According to the data obtained, the proportion of conserved G4 motifs in relation to all found G4 motifs in the compared set of mammalian species depended on the type of mammalian order and varied from 11 to 22%. Conserved G4 motifs were over-represented on the noncoding strand of *TERT* promotes, and their number, length, and localization varied across mammalian orders. The level of diversity in the conserved G4 motifs was also assessed using a block alignment approach: a statistically significant higher frequency of nucleotide substitutions in the conserved G4 motifs compared to the surrounding regions was confirmed only for the order *Primates*. This finding supports our hypothesis that G4 structures formed in oncogene promoters may act as mutagenic factors leading to genome instability in malignant cells due to interference with the DNA repair machinery.

## Figures and Tables

**Table 1:** Number of G4 motifs found in the coding and noncoding strands of the *TERT* promoter zones: core, proximal, and distal, for five orders of mammals with more than 10 species; all others are considered together.

Order of Mammals	Number of *TERT* Promoters Considered	*TERT* Promoters without G4 Motifs	0–180	181–500	501–1000	Total
			Coding/Noncoding Strands			
*Primates*	25	0	0/61 *	14/48	5/13	19/122
*Carnivora*	25	0	6/60	2/64	25/10 **	33/134
*Artiodactyla*	24	0	37/25	8/40*	33/22	78/87
*Chiroptera*	14	1	8/18	6/7	8/5	22/30
*Rodentia*	27	4	1/21 *	12/11	10/14	23/46
Other	26	1	8/48	11/55	8/26	27/129
Total	141	6	60/233	53/225	89/90	202/548

* Significant (*p* < 0.001) decrease in the number of G4 motifs on the coding strand compared to the noncoding strand. ** Significant (*p* < 0.001) decrease in the number of G4 motifs on the noncoding strand compared to the coding strand.

**Table 2:** Number of conserved G4 motifs found in the coding and noncoding strands of the *TERT* promoter zones: core, proximal, and distal, for five orders of mammals with more than 10 species.

Order of Mammals	Number of *TERT* Promoters Considered	0–180	181–500	501–1000	Total
		Coding/Noncoding Strands			
*Primates*	25	0/11	1/5	1/3	2/19
*Carnivora*	25	1/11	0/16	5/2	6/29
*Artiodactyla*	24	7/5	1/10	5/2	13/17
*Chiroptera*	14	2/2	0/2	2/0	4/4
*Rodentia*	27	1/3	1/0	1/2	3/5
Total	141	11/32	3/33	14/9	28/74

**Table 3:** Comparison of calculated nucleotide substitution densities in G4 loop-forming sequences and control sequences for five orders of mammals.

Order of Mammals	*p* Value	*p* Value Calculated Using the Holm-Bonferroni Correction	The Found Number of G4 Motifs in which the Density of Nucleotide Substitutions C↔T and G↔A is Higher than in the Control
*Primates*	0.003947	0.019735	16 out of 20 **
*Carnivora*	0.02381	0.095240	13 out of 35
*Artiodactyla*	0.2648	0.627600	14 out of 30
*Chiroptera*	0.7797 *	0.779700	2 out of 8
*Rodentia*	0.2092 *	0.627600	4 out of 8

* Only 8 conserved G4 motifs were found in both these orders; perhaps no significant differences in the considered parameters were found due to the small amount of data. ** We were unable to find a control for one of the conserved G4 motif, so it was excluded from consideration.

**Table 4:** Number of species per mammalian order with preserved *TERT* promoter region.

Order	Order Code	Species Number
*Rodentia*	Rod	27
*Carnivora*	Car	25
*Primates*	Pri	25
*Artiodactyla*	Art	24
*Chiroptera*	Chi	14
Less than 10 Species per Order		
*Perissodactyla*	Per	4
*Diprotodontia*	Dip	3
*Eulipotyphla*	Eul	3
*Afrosoricida*	Afr	2
*Lagomorpha*	Lag	2
*Monotremata*	Mon	2
*Pholidota*	Pho	2
*Cingulata*, *Didelphimorphia*, *Macroscelidea*, *Pilosa*, *Proboscidea*, *Sirenia*, *Tubulidentata*, *Dasyuromorphia*	Cin, Did, Mac, Pil, Pro, Sir, Tub, Das	1 per each order
total	20 orders	141

## Data Availability

Data associated with this manuscript are available in the Results, Materials and Methods and in the Supplementary Materials sections. Please contact the corresponding author to access the additional unpublished data.

## Supplementary Materials

The following supporting information can be downloaded at: https://www.mdpi.com/article/10.3390/life13071478/s1, Figure S1: Number and distances of G4 motifs from the transcription start site on the coding (blue columns) and noncoding (orange columns) strands of the 1000 bp TERT promoter region for orders of mammals; Figure S2: Taxonomic trees constructed for five orders of mammals; the number of species is given in brackets; Figure S3: Average number of G4 motifs per order of mammals; Figure S4: Overlap of conserved G4 motif in TERT promoter regions with binding sites of transcription factors Sp1 and Ets. Black arrow marks +1 nucleotide in the transcript according to the hTERT gene annotation. Conserved G-tracts are marked with black frames. Note that this motif is formed on a noncoding DNA strand; thus, it resembles cytosine tracts. Experimentally proven Sp1 recognition sites, known as Sp1 sites 1 and 2, are shown as blue bars. The core of the Ets recognition site is shown by the red bar. The Ets site is located between Sp1 1 and 2 sites. Conserved G4 motifs are found in 18 higher species from the families Hominidae, Hylobatidae and Cercopithecidae, which together form the superorder clade Laurasiatheria. In five evolutionary distant primates, an alternative conserved G4 motif may be detected. It includes lower left G-tract in the black frame and three other G-tracts that are aligned with the G-tracts of the conserved G4 motif in Laurasiatheria. Our procedure of promoter definition sometimes results in an inaccurate TSS determination. This is the case in the PriMAfas sequence. The 3′-terminal G-tract is not defined in it, probably due to this kind of error; Table S1: The mammalian species used in this study. All 141 species are presented with identifiers, and taxonomy information is taken from the NCBI Taxonomy database. Some terms may not coincide with those commonly used in evolutionary biology; Table S2: The number of reliable block alignments in TERT promoter regions per orders; Table S3: The number of nucleotide substitutions and their type in the G4 loop-forming sequences of the TERT promoter regions (in the coding and noncoding strands) for different orders of mammals.

## References

[1] Varizhuk A., Ischenko D., Tsvetkov V., Novikov R., Kulemin N., Kaluzhny D., Vlasenok M., Naumov V., Smirnov I., Pozmogova G. (2017). The expanding repertoire of G4 DNA structures. Biochimie.

[2] Neupane A., Chariker J.H., Rouchka E.C. (2023). Structural and functional classification of G-quadruplex families within the human genome. Genes.

[3] Dolinnaya N.G., Ogloblina A.M., Yakubovskaya M.G. (2016). Structure, properties, and biological relevance of the DNA and RNA G-quadruplexes: Overview 50 years after their discovery. Biochemistry.

[4] Akter J., Katai Y., Sultana P., Takenobu H., Haruta M., Sugino R.P., Mukae K., Satoh S., Wada T., Ohira M. (2021). Loss of p53 suppresses replication stress-induced DNA damage in ATRX-deficient neuroblastoma. Oncogenesis.

[5] Maffia A., Ranise C., Sabbioneda S. (2020). From R-loops to G-quadruplexes: Emerging new threats for the replication fork. Int. J. Mol. Sci..

[6] Rider S.D., Gadgil R.Y., Hitch D.C., Damewood F.J., Zavada N., Shanahan M., Alhawach V., Shrestha R., Shin-ya K., Leffak M. (2022). Stable G-quadruplex DNA structures promote replication-dependent genome instability. J. Biol. Chem..

[7] Damerla R.R., Knickelbein K.E., Strutt S., Liu F.-J., Wang H., Opresko P.L. (2012). Werner syndrome protein suppresses the formation of large deletions during the replication of human telomeric sequences. Cell Cycle.

[8] Khristich A.N., Mirkin S.M. (2020). On the wrong DNA track: Molecular mechanisms of repeat-mediated genome instability. J. Biol. Chem..

[9] Fleming A.M., Burrows C.J. (2021). Oxidative stress-mediated epigenetic regulation by G-quadruplexes. NAR Cancer.

[10] Stein M., Eckert K.A. (2021). Impact of G-quadruplexes and chronic inflammation on genome instability: Additive effects during Carcinogenesis. Genes.

[11] Cadet J., Douki T., Ravanat J.-L. (2008). Oxidatively generated damage to the guanine moiety of DNA: Mechanistic aspects and formation in cells. Acc. Chem. Res..

[12] Pavlova A.V., Kubareva E.A., Monakhova M.V., Zvereva M.I., Dolinnaya N.G. (2021). Impact of G-quadruplexes on the regulation of genome integrity, DNA damage and repair. Biomolecules.

[13] Makova K.D., Weissensteiner M.H. (2023). Noncanonical DNA structures are drivers of genome evolution. Trends Genet..

[14] Zvereva M.I., Shcherbakova D.M., Dontsova O.A. (2010). Telomerase: Structure, functions, and activity regulation. Biochemistry.

[15] Hasanau T.N., Pisarev E.P., Kisil O.V., Zvereva M.E. (2023). The *TERT* promoter: A key player in the fight for cancer cell immortality. Biochemistry.

[16] Pavlova A.V., Savitskaya V.Y., Dolinnaya N.G., Monakhova M.V., Litvinova A.V., Kubareva E.A., Zvereva M.I. (2022). G-quadruplex formed by the promoter region of the *hTERT* gene: Structure-driven effects on DNA mismatch repair functions. Biomedicines.

[17] Autexier C., Lue N.F. (2006). The structure and function of telomeraser transcriptase. Annu. Rev. Biochem..

[18] Hon J., Martínek T., Zendulka J., Lexa M. (2017). pqsfinder: An exhaustive and imperfection-tolerant search tool for potential quadruplex-forming sequences in *R*. Bioinformatics.

[19] Bedrat A., Lacroix L., Mergny J.-L. (2016). Re-evaluation of G-quadruplex propensity with G4Hunter. Nucleic Acids Res..

[20] Kikin O., D’Antonio L., Bagga P.S. (2006). QGRS Mapper: A web-based server for predicting G-quadruplexes in nucleotide sequences. Nucleic Acids Res..

[21] Fisher R.A. (2006). Statistical Methods for Research Workers.

[22] Liu R., Xing M. (2016). *TERT* promoter mutations in thyroid cancer. Endocr. Relat. Cancer.

[23] Guo Y., Chen Y., Zhang L., Ma L., Jiang K., Yao G., Zhu L. (2022). *TERT* promoter mutations and telomerase in melanoma. J. Oncol..

[24] Hasanau T., Pisarev E., Kisil O., Nonoguchi N., Le Calvez-Kelm F., Zvereva M. (2022). Detection of *TERT* promoter mutations as a prognostic biomarker in gliomas: Methodology, prospects, and advances. Biomedicines.

[25] Zvereva M., Pisarev E., Hosen I., Kisil O., Matskeplishvili S., Kubareva E., Kamalov D., Tivtikyan A., Manel A., Vian E. (2020). Activating telomerase *TERT* promoter mutations and their application for the detection of bladder cancer. Int. J. Mol. Sci..

[26] McKinney A.M., Mathur R., Stevers N.O., Molinaro A.M., Chang S.M., Phillips J.J., Costello J.F. (2022). GABP couples oncogene signaling to telomere regulation in *TERT* promoter mutant cancer. Cell Rep..

[27] Yuan X., Dai M., Xu D. (2020). *TERT* promoter mutations and GABP transcription factors in carcinogenesis: More foes than friends. Cancer Lett..

[28] Vinagre J., Almeida A., Pópulo H., Batista R., Lyra J., Pinto V., Coelho R., Celestino R., Prazeres H., Lima L. (2013). Frequency of *TERT* promoter mutations in human cancers. Nat. Commun..

[29] Vincze O., Colchero F., Lemaître J.-F., Conde D.A., Pavard S., Bieuville M., Urrutia A.O., Ujvari B., Boddy A.M., Maley C.C. (2022). Cancer risk across mammals. Nature.

[30] Huppert J.L., Balasubramanian S. (2007). G-quadruplexes in promoters throughout the human genome. Nucleic Acids Res..

[31] Seluanov A., Gladyshev V.N., Vijg J., Gorbunova V. (2018). Mechanisms of cancer resistance in long-lived mammals. Nat. Rev. Cancer.

[32] Jia L., Mao Y., Ji Q., Dersh D., Yewdell J.W., Qian S.-B. (2020). Decoding mRNA translatability and stability from the 5′ UTR. Nat. Struct. Mol. Biol..

[33] Pavlova A.V., Monakhova M.V., Ogloblina A.M., Andreeva N.A., Laptev G.Y., Polshakov V.I., Gromova E.S., Zvereva M.I., Yakubovskaya M.G., Oretskaya T.S. (2020). Responses of DNA mismatch repair proteins to a stable G-quadruplex embedded into a DNA duplex structure. Int. J. Mol. Sci..

