# Supplementary material for: Conserved G-Quadruplex-Forming Sequences in Mammalian TERT Promoters and Their Effect on Mutation Frequency
Source: Life (Basel). 2023 Jun 29;13(7):1478. doi: 10.3390/life13071478 (PMC10381784; doi:10.3390/life13071478)
Supplement: Supplementary file 1 [file life-13-01478-s001.zip › Supplementary Panova.pdf]

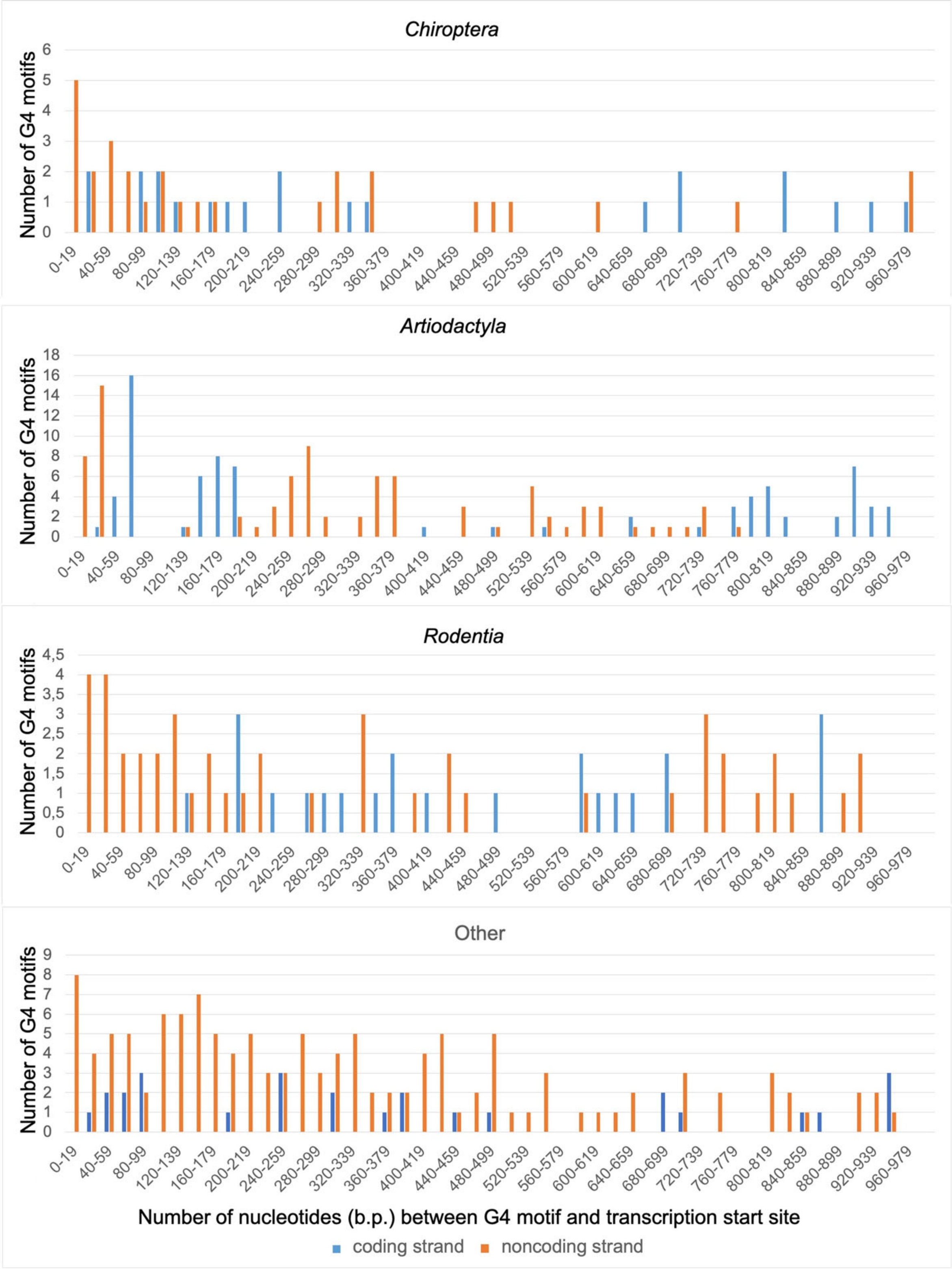

Figure S1. Number and distances of G4 motifs from the transcription start site on the coding (blue columns) and noncoding (orange columns) strands of the 1000-bp *TERT* promoter region for different orders of mammals.

1)*Primates*

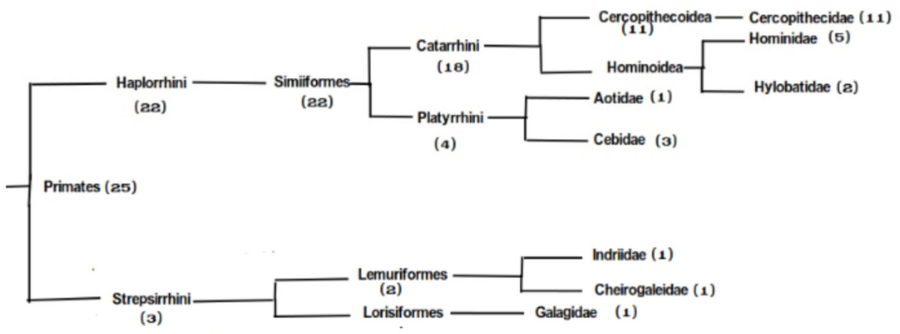

2) *Artiodactyla*

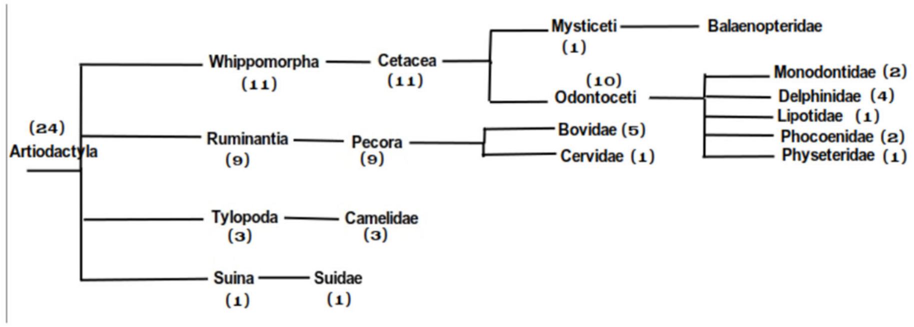

3)*Carnivora*

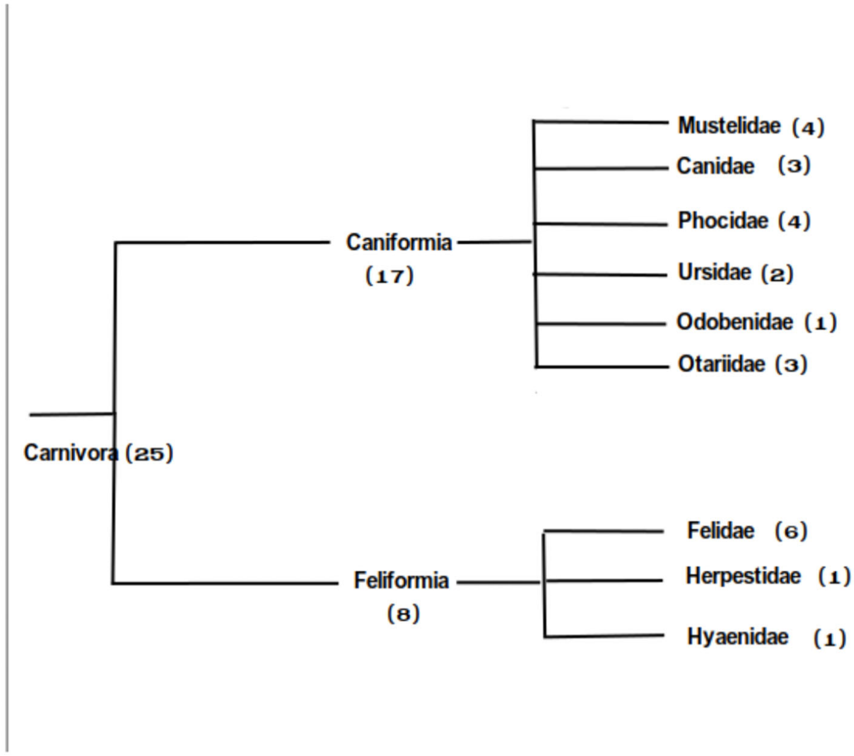

4)*Rodentia*

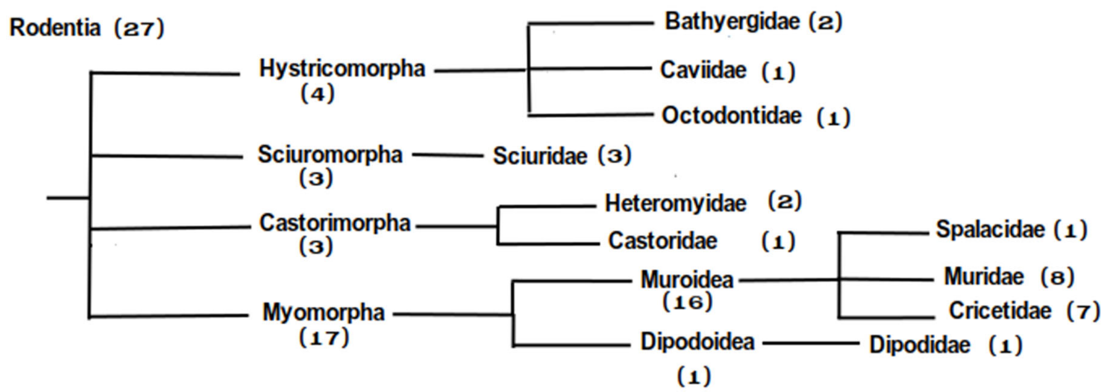

5) *Chiroptera*

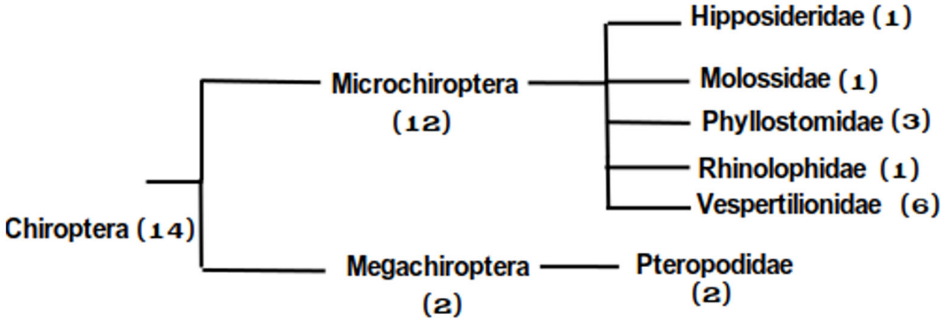

Figure S2. Taxonomical trees constructed for five orders of mammals; the number of species is given in brackets.

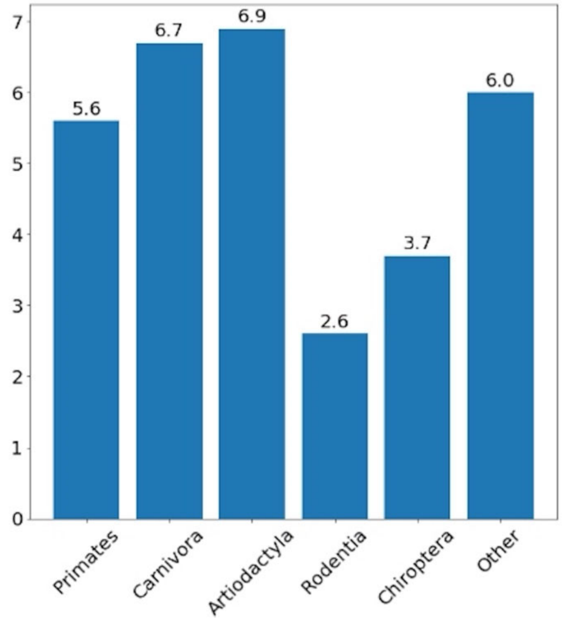

Figure S3. Average number of G4 motifs per species for different orders of mammals.

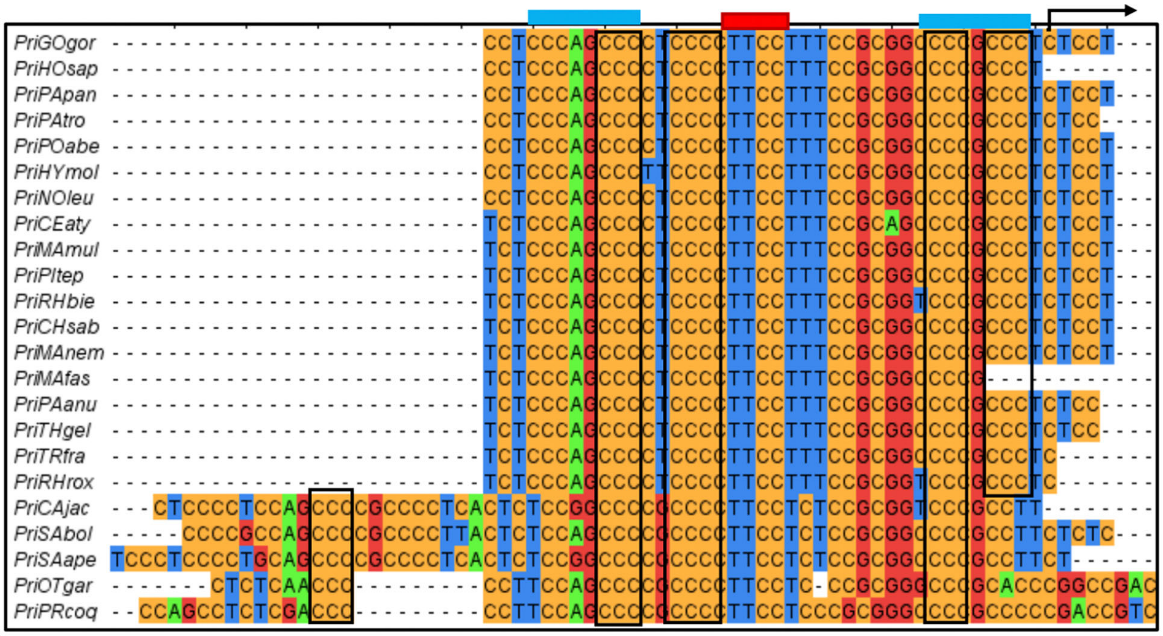

Figure S4. Overlap of conserved G4 motif in *TERT* promoter regions with binding sites of transcription factors Sp1 and Ets. Black arrow marks +1 nucleotide in the transcript according to the *hTERT* gene annotation. Conserved G-tracts are marked with black frames. Note that this motif is formed on a noncoding DNA strand, so it looks like cytosine tracts. Experimentally proven Sp1 recognition sites, known as Sp1 sites 1 and 2, are shown as blue bars. The core of Ets recognition site is shown by the red bar. The Ets site is located between Sp1 1 and 2 sites. Conserved G4 motifs are found in 18 higher species from the families *Hominidae*, *Hylobatidae* and *Cercopithecidae*, which together form the superorder clade *Laurasiatheria*. In five evolutionary distant primates, an alternative conserved G4 motif may be detected. It includes lower left G-tract in black frame and three other G-tracts that are aligned with the G-tracts of the conserved G4 motif in *Laurasiatheria.* Our procedure of promoter definition sometime results in an inaccurate TSS determination. This is the case in the PriMAfas sequence. The 3'-terminal G-tract is not defined in it, probably due to this kind of error.

Table S1. The mammalian species used in this study. All 141 species are presented with identificators and taxonomy information taken from the NCBI Taxonomy database. Some terms may not coincides with those commonly used in evolutionary biology.

Scientific name	species code	AC	order code	Number of species per order	subclass, clade, superorder, clade	order	Between order and family	family	subfamily	genus
Castor canadensis	rodCAcan	NW_017872232	Rod	27	Th-Eu-Eua-Gli	Rodentia	Castorimorpha	Castoridae		Castor
Dipodomys spectabilis	rodDIspe	NW_024870856	Rod	27	Th-Eu-Eua-Gli	Rodentia	Castorimorpha	Heteromyidae	Dipodomyinae	Dipodomys
Dipodomys ordii	rodDIord	NW_012267237	Rod	27	Th-Eu-Eua-Gli	Rodentia	Castorimorpha	Heteromyidae	Dipodomyinae	Dipodomys
Fukomys damarensis	rodFUdam	NW_022900913	Rod	27	Th-Eu-Eua-Gli	Rodentia	Hystricomorpha	Bathyergidae		Fukomys
Heterocephalus glaber	rodHEgla	NW_004624751	Rod	27	Th-Eu-Eua-Gli	Rodentia	Hystricomorpha	Bathyergidae		Heterocephalus
Cavia porcellus	rodCApor	NT_176269	Rod	27	Th-Eu-Eua-Gli	Rodentia	Hystricomorpha	Caviidae		Cavia
Octodon degus	rodOCdeg	NW_004524584	Rod	27	Th-Eu-Eua-Gli	Rodentia	Hystricomorpha	Octodontidae		Octodon
Jaculus jaculus	rodJAjac	NC_059121	Rod	27	Th-Eu-Eua-Gli	Rodentia	Myo-Dip	Dipodidae	Dipodinae	Jaculus
Microtus oregoni	rodMIore	NW_024543207	Rod	27	Th-Eu-Eua-Gli	Rodentia	Myo-Mur	Cricetidae	Arvicolinae	Microtus
Microtus ochrogaster	rodMIoch	NC_022021	Rod	27	Th-Eu-Eua-Gli	Rodentia	Myo-Mur	Cricetidae	Arvicolinae	Microtus

Cricetulus griseus	rodCRgri	NC_048595	Rod	27	Th-Eu-Eua-Gli	Rodentia	Myo-Mur	Cricetidae	Cricetinae	Cricetulus
Mesocricetus auratus	rodMEaur	NW_024429198	Rod	27	Th-Eu-Eua-Gli	Rodentia	Myo-Mur	Cricetidae	Cricetinae	Mesocricetus
Onychomys torridus	rodONtor	NC_050457	Rod	27	Th-Eu-Eua-Gli	Rodentia	Myo-Mur	Cricetidae	Neotominae	Onychomys
Peromyscus leucopus	rodPEleu	NC_051079	Rod	27	Th-Eu-Eua-Gli	Rodentia	Myo-Mur	Cricetidae	Neotominae	Peromyscus
Peromyscus maniculatus bairdii	rodPEman	NC_056022	Rod	27	Th-Eu-Eua-Gli	Rodentia	Myo-Mur	Cricetidae	Neotominae	Peromyscus maniculatus
Meriones unguiculatus	rodMEung	NW_018657810	Rod	27	Th-Eu-Eua-Gli	Rodentia	Myo-Mur	Muridae	Gerbillinae	Meriones
Arvicanthis niloticus	rodARnil	NC_047676	Rod	27	Th-Eu-Eua-Gli	Rodentia	Myo-Mur	Muridae	Murinae	Arvicanthis
Grammomys surdaster	rodGRsur	NW_021605262	Rod	27	Th-Eu-Eua-Gli	Rodentia	Myo-Mur	Muridae	Murinae	Grammomys
Mastomys coucha	rodMAcou	NW_022196914	Rod	27	Th-Eu-Eua-Gli	Rodentia	Myo-Mur	Muridae	Murinae	Mastomys
Mus pahari	rodMUpah	NC_034600	Rod	27	Th-Eu-Eua-Gli	Rodentia	Myo-Mur	Muridae	Murinae	Mus Coelomys
Mus musculus	rodMUmus	NC_000079	Rod	27	Th-Eu-Eua-Gli	Rodentia	Myo-Mur	Muridae	Murinae	Mus Mus
Mus caroli	rodMUcar	NC_034582	Rod	27	Th-Eu-Eua-Gli	Rodentia	Myo-Mur	Muridae	Murinae	Mus Mus
Rattus norvegicus	rodRAnor	NC_051336	Rod	27	Th-Eu-Eua-Gli	Rodentia	Myo-Mur	Muridae	Murinae	Rattus
Nannospalax galili	rodNAgal	NW_008355380	Rod	27	Th-Eu-Eua-Gli	Rodentia	Myo-Mur	Spalacidae	Spalacinae	Nannospalax
Ictidomys tridecemlineatus	rodICtri	NW_024407213	Rod	27	Th-Eu-Eua-Gli	Rodentia	Sciuromorpha	Sciuridae	Xerinae	Ictidomys

Marmota flaviventris	rodMAfla	NW_023144708	Rod	27	Th-Eu-Eua-Gli	Rodentia	Sciuromorpha	Sciuridae	Xerinae	Marmota
Urocitellus parryii	rodURpar	NW_020540633	Rod	27	Th-Eu-Eua-Gli	Rodentia	Sciuromorpha	Sciuridae	Xerinae	Urocitellus
Cercocebus atys	priCEaty	NW_012002678	Pri	25	Th-Eu-Lau	Primates	Haplo-Simii-Cat-Cer	Cercopithecidae	Cercopithecinae	Cercocebus
Chlorocebus sabaeus	priCHsab	NW_023666054	Pri	25	Th-Eu-Lau	Primates	Haplo-Simii-Cat-Cer	Cercopithecidae	Cercopithecinae	Chlorocebus
Macaca nemestrina	priMAnem	NW_012012689	Pri	25	Th-Eu-Lau	Primates	Haplo-Simii-Cat-Cer	Cercopithecidae	Cercopithecinae	Macaca
Macaca mulatta	priMAmul	NC_041759	Pri	25	Th-Eu-Lau	Primates	Haplo-Simii-Cat-Cer	Cercopithecidae	Cercopithecinae	Macaca
Macaca fascicularis	priMAfas	NC_052260	Pri	25	Th-Eu-Lau	Primates	Haplo-Simii-Cat-Cer	Cercopithecidae	Cercopithecinae	Macaca
Papio anubis	priPAanu	NC_044980	Pri	25	Th-Eu-Lau	Primates	Haplo-Simii-Cat-Cer	Cercopithecidae	Cercopithecinae	Papio
Theropithecus gelada	priTHgel	NC_037673	Pri	25	Th-Eu-Lau	Primates	Haplo-Simii-Cat-Cer	Cercopithecidae	Cercopithecinae	Theropithecus
Piliocolobus tephrosceles	priPItep	NC_045437	Pri	25	Th-Eu-Lau	Primates	Haplo-Simii-Cat-Cer	Cercopithecidae	Colobinae	Piliocolobus
Rhinopithecus roxellana	priRHrox	NC_044551	Pri	25	Th-Eu-Lau	Primates	Haplo-Simii-Cat-Cer	Cercopithecidae	Colobinae	Rhinopithecus
Rhinopithecus bieti	priRHbie	NW_016814059	Pri	25	Th-Eu-Lau	Primates	Haplo-Simii-Cat-Cer	Cercopithecidae	Colobinae	Rhinopithecus
Trachypithecus francoisi	priTRfra	NW_022681465	Pri	25	Th-Eu-Lau	Primates	Haplo-Simii-Cat-Cer	Cercopithecidae	Colobinae	Trachypithecus
Gorilla gorilla gorilla	priGOgor	NC_044619	Pri	25	Th-Eu-Lau	Primates	Haplo-Simii-Cat-Hom	Hominidae	Homininae	Gorilla gorilla

Homo sapiens	priHOsap	NC_000005	Pri	25	Th-Eu-Lau	Primates	Haplo-Simii-Cat-Hom	Hominidae	Homininae	Homo
Pan troglodytes	priPAtro	NC_036884	Pri	25	Th-Eu-Lau	Primates	Haplo-Simii-Cat-Hom	Hominidae	Homininae	Pan
Pan paniscus	priPApan	NC_048244	Pri	25	Th-Eu-Lau	Primates	Haplo-Simii-Cat-Hom	Hominidae	Homininae	Pan
Pongo abelii	priPOabe	NC_036908	Pri	25	Th-Eu-Lau	Primates	Haplo-Simii-Cat-Hom	Hominidae	Ponginae	Pongo
Hylobates moloch	priHYmol	NW_022611659	Pri	25	Th-Eu-Lau	Primates	Haplo-Simii-Cat-Hom	Hylobatidae		Hylobates
Nomascus leucogenys	priNOleu	NC_044386	Pri	25	Th-Eu-Lau	Primates	Haplo-Simii-Cat-Hom	Hylobatidae		Nomascus
Aotus nancymaae	priAOnan	NW_018503740	Pri	25	Th-Eu-Lau	Primates	Haplo-Simii-Plat	Aotidae		Aotus
Callithrix jacchus	priCAjac	NC_048384	Pri	25	Th-Eu-Lau	Primates	Haplo-Simii-Plat	Cebidae	Callitrichinae	Callithrix Callithrix
Sapajus apella	priSAape	NW_022436951	Pri	25	Th-Eu-Lau	Primates	Haplo-Simii-Plat	Cebidae	Cebinae	Sapajus
Saimiri boliviensis	priSAbol	NW_024100917	Pri	25	Th-Eu-Lau	Primates	Haplo-Simii-Plat	Cebidae	Saimiriinae	Saimiri
Microcebus murinus	priMImur	NC_033671	Pri	25	Th-Eu-Lau	Primates	Strep-lemur	Cheirogaleidae		Microcebus
Propithecus coquereli	priPRcoq	NW_012154474	Pri	25	Th-Eu-Lau	Primates	Strep-lemur	Indriidae		Propithecus
Otolemur garnettii	priOTgar	NW_003852621	Pri	25	Th-Eu-Lau	Primates	Strep-Loris	Galagidae		Otolemur
Canis lupus dingo	carCAlup	NC_051838	Car	25	Th-Eu-Lau	Carnivora	Caniformia	Canidae	Canis	Canis lupus

Canis lupus familiaris	carCAlup	NC_051838	Car	25	Th-Eu-Lau	Carnivora	Caniformia	Canidae	Canis	Canis lupus
Vulpes lagopus	carVUlag	NC_054840	Car	25	Th-Eu-Lau	Carnivora	Caniformia	Canidae		Vulpes
Enhydra lutris kenyoni	carENlut	NW_019154116	Car	25	Th-Eu-Lau	Carnivora	Caniformia	Mustelidae	Lutrinae	Enhydra lutris
Lontra canadensis	carLOcan	NW_022631016	Car	25	Th-Eu-Lau	Carnivora	Caniformia	Mustelidae	Lutrinae	Lontra
Mustela erminea	carMUerm	NC_045616	Car	25	Th-Eu-Lau	Carnivora	Caniformia	Mustelidae	Mustelinae	Mustela
Mustela putorius furo	carMUput	NW_025421284	Car	25	Th-Eu-Lau	Carnivora	Caniformia	Mustelidae	Mustelinae	Mustela putorius
Odobenus rosmarus divergens	carODros	NW_004450831	Car	25	Th-Eu-Lau	Carnivora	Caniformia	Odobenidae	Odobenus	Odobenus rosmarus
Callorhinus ursinus	carCAurs	NW_020312816	Car	25	Th-Eu-Lau	Carnivora	Caniformia	Otariidae		Callorhinus
Eumetopias jubatus	carEUjub	NW_020998780	Car	25	Th-Eu-Lau	Carnivora	Caniformia	Otariidae		Eumetopias
Zalophus californianus	carZAcal	NC_045599	Car	25	Th-Eu-Lau	Carnivora	Caniformia	Otariidae		Zalophus
Halichoerus grypus	carHAgry	NW_023400076	Car	25	Th-Eu-Lau	Carnivora	Caniformia	Phocidae		Halichoerus
Mirounga leonina	carMIleo	NW_023270865	Car	25	Th-Eu-Lau	Carnivora	Caniformia	Phocidae		Mirounga
Neomonachus schauinslandi	carNEsch	NC_058409	Car	25	Th-Eu-Lau	Carnivora	Caniformia	Phocidae		Neomonachus
Phoca vitulina	carPHvit	NW_022589720	Car	25	Th-Eu-Lau	Carnivora	Caniformia	Phocidae		Phoca
Ursus arctos horribilis	carURarc	NW_025929695	Car	25	Th-Eu-Lau	Carnivora	Caniformia	Ursidae	Ursus	Ursus arctos

Ursus maritimus	carURmar	NW_024423897	Car	25	Th-Eu-Lau	Carnivora	Caniformia	Ursidae		Ursus
Acinonyx jubatus	carACjub	NW_020834736	Car	25	Th-Eu-Lau	Carnivora	Feliformia	Felidae	Acinonychinae	Acinonyx
Felis catus	carFEcat	NC_058368	Car	25	Th-Eu-Lau	Carnivora	Feliformia	Felidae	Felinae	Felis
Lynx canadensis	carLYcan	NC_044303	Car	25	Th-Eu-Lau	Carnivora	Feliformia	Felidae	Felinae	Lynx
Puma yagouaroundi	carPUyag	NW_024412377	Car	25	Th-Eu-Lau	Carnivora	Feliformia	Felidae	Felinae	Puma
Puma concolor	carPUcon	NW_020339444	Car	25	Th-Eu-Lau	Carnivora	Feliformia	Felidae	Felinae	Puma
Panthera tigris altaica	carPAtig	NC_056660	Car	25	Th-Eu-Lau	Carnivora	Feliformia	Felidae	Pantherinae	Panthera tigris
Suricata suricatta	carSUsur	NC_043705	Car	25	Th-Eu-Lau	Carnivora	Feliformia	Herpestidae		Suricata
Hyaena hyaena	carHYhya	NW_024080766	Car	25	Th-Eu-Lau	Carnivora	Feliformia	Hyaenidae		Hyaena
Camelus ferus	artCAfer	NC_045698	Art	23	Th-Eu-Lau	Artiodactyla	Tylopoda	Camelidae		Camelus
Camelus dromedarius	artCAdro	NC_044513	Art	23	Th-Eu-Lau	Artiodactyla	Tylopoda	Camelidae		Camelus
Vicugna pacos	artVIpac	NW_021964160	Art	23	Th-Eu-Lau	Artiodactyla	Tylopoda	Camelidae		Vicugna
Bison bison bison	artBIbis	NW_011494949	Art	23	Th-Eu-Lau	Artiodactyla	Ru-Pec	Bovidae	Bovinae	Bison bison
Bos indicus	artBOind	NC_032669	Art	23	Th-Eu-Lau	Artiodactyla	Ru-Pec	Bovidae	Bovinae	Bos
Bos taurus	artBOtau	NC_037347	Art	23	Th-Eu-Lau	Artiodactyla	Ru-Pec	Bovidae	Bovinae	Bos
Bubalus bubalis	artBUbub	NC_059175	Art	23	Th-Eu-Lau	Artiodactyla	Ru-Pec	Bovidae	Bovinae	Bubalus
Capra hircus	artCAhir	NC_030827	Art	23	Th-Eu-Lau	Artiodactyla	Ru-Pec	Bovidae	Caprinae	Capra
Ovis aries	artOVari	NC_056069	Art	23	Th-Eu-Lau	Artiodactyla	Ru-Pec	Bovidae	Caprinae	Ovis
Oryx dammah	artORdam	NW_024070204	Art	23	Th-Eu-Lau	Artiodactyla	Ru-Pec	Bovidae	Hippotraginae	Oryx
Odocoileus virginianus texanus	artODvir	NW_018332784	Art	23	Th-Eu-Lau	Artiodactyla	Ru-Pec	Cervidae	Odocoileinae	Odocoileus virginianus

Sus scrofa	artSUscr	NC_010458	Art	23	Th-Eu-Lau	Artiodactyla	Suina	Suidae		Sus
Balaenoptera musculus	artBAmus	NC_045787	Art	23	Th-Eu-Lau	Artiodactyla	Whi-Cet-Mys	Balaenopteridae		Balaenoptera
Globicephala melas	artGLmel	NW_022134865	Art	23	Th-Eu-Lau	Artiodactyla	Whi-Cet-odo	Delphinidae		Globicephala
Lagenorhynchus obliquidens	artLAobl	NW_020837973	Art	23	Th-Eu-Lau	Artiodactyla	Whi-Cet-odo	Delphinidae		Lagenorhynchus
Orcinus orca	artORorc	NC_064561	Art	23	Th-Eu-Lau	Artiodactyla	Whi-Cet-odo	Delphinidae		Orcinus
Tursiops truncatus	artTUtru	NC_047036	Art	23	Th-Eu-Lau	Artiodactyla	Whi-Cet-odo	Delphinidae		Tursiops
Lipotes vexillifer	artLIvex	NW_006799734	Art	23	Th-Eu-Lau	Artiodactyla	Whi-Cet-odo	Lipotidae		Lipotes
Delphinapterus leucas	artDEleu	NW_022098008	Art	23	Th-Eu-Lau	Artiodactyla	Whi-Cet-odo	Monodontidae		Delphinapterus
Monodon monoceros	artMOmon	NW_021703768	Art	23	Th-Eu-Lau	Artiodactyla	Whi-Cet-odo	Monodontidae		Monodon
Neophocaena asiaeorientalis asiaeorientalis	artNEasi	NW_020175679	Art	23	Th-Eu-Lau	Artiodactyla	Whi-Cet-odo	Phocoenidae		Neophocaena asiaeorientalis
Phocoena sinus	artPHsin	NC_045765	Art	23	Th-Eu-Lau	Artiodactyla	Whi-Cet-odo	Phocoenidae		Phocoena
Physeter catodon	artPHcat	NC_041221	Art	23	Th-Eu-Lau	Artiodactyla	Whi-Cet-odo	Physeteridae		Physeter
Hipposideros armiger	chiHIarm	NW_017731935	Chi	14	Th-Eu-Lau	Chiroptera	Microchiroptera	Hipposideridae		Hipposideros
Molossus molossus	chiMOmol	NW_023425366	Chi	14	Th-Eu-Lau	Chiroptera	Microchiroptera	Molossidae		Molossus
Desmodus rotundus	chiDErot	NW_020093731	Chi	14	Th-Eu-Lau	Chiroptera	Microchiroptera	Phyllostomidae	Desmodontinae	Desmodus

Phyllostomus discolor	chiPHdis	NC_040905	Chi	14	Th-Eu-Lau	Chiroptera	Microchiroptera	Phyllostomidae	Phyllostominae	Phyllostomus
Sturnira hondurensis	chiSThon	NW_023521217	Chi	14	Th-Eu-Lau	Chiroptera	Microchiroptera	Phyllostomidae	Stenodermatinae	Sturnira
Rhinolophus ferrumequinum	chiRHfer	NC_046290	Chi	14	Th-Eu-Lau	Chiroptera	Microchiroptera	Rhinolophidae	Rhinolophinae	Rhinolophus
Eptesicus fuscus	chiEPfus	NW_007370656	Chi	14	Th-Eu-Lau	Chiroptera	Microchiroptera	Vespertilionidae		Eptesicus
Myotis davidii	chiMYdav	NW_006289813	Chi	14	Th-Eu-Lau	Chiroptera	Microchiroptera	Vespertilionidae		Myotis
Myotis brandtii	chiMYbra	NW_005353967	Chi	14	Th-Eu-Lau	Chiroptera	Microchiroptera	Vespertilionidae		Myotis
Myotis lucifugus	chiMYluc	NW_005871048	Chi	14	Th-Eu-Lau	Chiroptera	Microchiroptera	Vespertilionidae		Myotis
Myotis myotis	chiMYmyo	NW_023416357	Chi	14	Th-Eu-Lau	Chiroptera	Microchiroptera	Vespertilionidae		Myotis
Pipistrellus kuhlii	chiPIkuh	NW_023425540	Chi	14	Th-Eu-Lau	Chiroptera	Microchiroptera	Vespertilionidae		Pipistrellus
Pteropus giganteus	chiPTgig	NW_024351073	Chi	14	Th-Eu-Lau	Chiroptera	Megachiroptera	Pteropodidae	Pteropodinae	Pteropus
Rousettus aegyptiacus	chiROaeg	NW_023416311	Chi	14	Th-Eu-Lau	Chiroptera	Megachiroptera	Pteropodidae	Pteropodinae	Rousettus
Equus przewalskii	perEQprz	NW_007673864	Per	4	Th-Eu-Lau	Perissodactyla		Equidae		Equus
Equus caballus	perEQcab	NC_009164	Per	4	Th-Eu-Lau	Perissodactyla		Equidae		Equus
Equus asinus	perEQasi	NC_052186	Per	4	Th-Eu-Lau	Perissodactyla		Equidae		Equus
Ceratotherium simum simum	perCEsim	NW_004454243	Per	4	Th-Eu-Lau	Perissodactyla		Rhinocerotidae	Ceratotherium	Ceratotherium simum
Erinaceus europaeus	eulERЕur	NW_006804048	Eul	3	Th-Eu-Lau	Eulipotyphla		Erinaceidae	Erinaceinae	Erinaceus
Sorex araneus	eulSOara	NW_004546046	Eul	3	Th-Eu-Lau	Eulipotyphla		Soricidae	Soricinae	Sorex
Talpa occidentalis	eulTAocc	NW_023604972	Eul	3	Th-Eu-Lau	Eulipotyphla		Talpidae		Talpa

Trichosurus vulpecula	dipTRvul	NC_050573	Dip	3	Th-Met	Diprotodontia		Phalangeridae		Trichosurus
Phascolarctos cinereus	dipPHcin	NW_018344251	Dip	3	Th-Met	Diprotodontia		Phascolarctidae		Phascolarctos
Vombatus ursinus	dipVOurs	NW_020954569	Dip	3	Th-Met	Diprotodontia		Vombatidae		Vombatus
Orycteropus afer afer	tubORafe	NW_006921890	Tub	2	Th-Eu-Afro	Tubulidentata		Orycteropodidae		Orycteropus
Orycteropus afer	tubORafe	NW_006921890	Tub	2	Th-Eu-Afro	Tubulidentata		Orycteropodidae		Orycteropus
Manis pentadactyla	phoMApen	NW_023454126	Pho	2	Th-Eu-Lau	Pholidota		Manidae		Manis
Manis javanica	phoMAjav	NW_023436000	Pho	2	Th-Eu-Lau	Pholidota		Manidae		Manis
Ornithorhynchus anatinus	monORana	NC_041751	Mon	2	Prototheria	Monotremata		Ornithorhynchidae		Ornithorhynchus
Tachyglossus aculeatus	monTAacu	NC_052099	Mon	2	Prototheria	Monotremata		Tachyglossidae		Tachyglossus
Ochotona curzoniae	lagOCcur	NW_024465582	Lag	2	Th-Eu-Eua-Gli	Lagomorpha		Ochotonidae		Ochotona
Ochotona princeps	lagOCpri	NC_050546	Lag	2	Th-Eu-Eua-Gli	Lagomorpha		Ochotonidae		Ochotona
Chrysochloris asiatica	afrCHasi	NW_006408713	Afr	2	Th-Eu-Afro	Afrosoricida		Chrysochloridae	Chrysochlorinae	Chrysochloris
Echinops telfairi	afrECtel	NW_022111373	Afr	2	Th-Eu-Afro	Afrosoricida		Tenrecinae		Echinops
Trichechus manatus latirostris	sirTRman	NW_004444008	Sir	1	Th-Eu-Afro	Sirenia		Trichechidae		Trichechus manatus

Loxodonta africana	proLOafr	NW_003573427	Pro	1	Th-Eu	Proboscidea		Elephantidae		Loxodonta
Choloepus didactylus	pilCHdid	NC_051317	Pil	1	Th-Eu-Xen	Pilosa	Folivora	Megalonychidae		Choloepus
Elephantulus edwardii	macELedw	NW_006399875	Mac	1	Th-Eu-Afro	Macroscelidea		Macroscelididae		Elephantulus
Monodelphis domestica	didMOdom	NC_008803	Did	1	Th-Met	Didelphimorphia		Didelphidae	Didelphinae	Monodelphis
Sarcophilus harrisii	dasSAhar	NC_045426	Das	1	Th-Met	Dasyuromorphia		Dasyuridae		Sarcophilus
Dasypus novemcinctus	cinDAnov	NW_004481457	Cin	1	Th-Eu-Xen	Cingulata		Dasypodidae		Dasypus

Table S2. The number of reliable block alignments in *TERT* promoter regions per orders.

Order of mammals	*Primates*	*Artiodactyla*	*Carnivora*	*Rodentia*	*Chiroptera*
Number of aligned blocks	12	18	14	23	11
Number of nucleotides in reliable block* alignments (% of all nucleotides in promoters)	19148 (77%)	19641 (82%)	22044 (88%)	14041 (52%)	10111 (72%)
block's names**	h18x413<br>h18x141<br>h14x111<br>h14x107<br>h11x123<br>h5x139<br>h4x183<br>h4x145<br>h3x708<br>h2x111<br>h2x106<br>h2x103	h13x114<br>h11x275<br>h11x152<br>h11x149<br>h10x153<br>h9x115<br>h9x115n1<br>h8x262<br>h7x157<br>h7x117<br>h5x119<br>h4x109<br>h3x722<br>h3x168<br>h2x129<br>h2x125	h10x119<br>h8x680<br>h8x102<br>h7x183<br>h7x162<br>h7x112<br>h6x427<br>h6x135<br>h4x707<br>h3x959<br>h2x690<br>h2x195<br>h2x145<br>h2x131	h10x102<br>h4x296<br>h4x205<br>h4x174<br>h3x999<br>h3x334<br>h3x193<br>h3x165<br>h3x153<br>h3x112<br>h3x109<br>h3x105<br>h3x105n1<br>h3x100<br>h3x100n1<br>h2x356<br>h2x222<br>h2x199<br>h2x166<br>h2x151<br>h2x131<br>h2x120<br>h2x103	h5x765<br>h3x159<br>h3x133<br>h3x120<br>h2x1006<br>h2x697<br>h2x257<br>h2x157<br>h2x154<br>h2x132<br>h2x122

* The number is computed as the sum of products of the block length on the number of species in the block. This product exceeds the number of nucleotides in the block by the number of gaps. The number of gaps in the block is limited because of the requirement: 80% or more identical positions in a reliable block alignment.
** Letter "h" denotes reliable block alignments, which include not all species of the order. The number of species within the block is written between characters "h" and "x". "x" is the delimiter. The length of the block is after "x". Letters "n1" are added to distinguish blocks with identical core names.

The set of species within a block is mainly matched with taxonomy divisions of various ranges. There are exceptions. The number of exceptions: *Primates*: 2, *Carnivora*: 1, *Artiodactyla*: 4, *Rodentia*: 11, *Chiroptera*:5. We conclude that blocks of reliable alignments cover more than half of promoter regions within each order.

Table S3. The number of nucleotide substitutions and their type in the G4 loop-forming sequences of the *TERT* promoter regions (in the coding and noncoding strands) for different orders of mammals.

Order	Type of nucleotide substitutions	Number of nucleotide substitutions	
		Coding strand	Noncoding strand
Artiodactyla	A↔G	9	8
	C↔T	13	16
	C↔G	4	5
	gap↔C	3	6
	A↔C	2	3
	gap↔G	1	0
	G↔T	3	1
	A↔T	1	0
	gap↔T	1	0
Rodentia	A↔G	0	3
	C↔T	1	2
	C↔G	1	1
	gap↔C	0	2
	A↔C	2	0
	gap↔A	0	4
	G↔T	0	1
	A↔T	0	0
Carnivora	A↔G	2	4
	G↔T	2	4
	C↔T	6	17
	A↔C	1	6
	gap↔G	1	4
	gap↔T	1	4
	C↔G	2	14
	gap↔C	0	10
	A↔T	2	1
	gap↔A	0	1
Primates	A↔G	1	10
	G↔T	0	4
	C↔T	0	17
	C↔G	0	9
	A↔C	0	6
	gap↔C	0	6
	gap↔G	0	1
	A↔T	0	1
	gap↔A	0	2
	gap↔T	0	2
Chiroptera	C↔T	4	1
	G↔T	0	1
	gap↔G	0	1
	A↔G	2	2
	gap↔A	0	1
